# Targeted photo-chemo therapy of malignancy on the chest wall while cardiopulmonary avoidance based on Fe_3_O_4_@ZnO nanocomposites

**DOI:** 10.18632/oncotarget.9123

**Published:** 2016-05-02

**Authors:** Haijun Zhang, Liting Guo, Shuang Ding, Jian Xiong, Baoan Chen

**Affiliations:** ^1^ Department of Oncology, Zhongda Hospital, School of Medicine, Southeast University, Nanjing, People's Republic of China; ^2^ Department of Hematology, Zhongda Hospital, School of Medicine, Southeast University, Nanjing, People's Republic of China

**Keywords:** Fe_3_O_4_@ZnO nanocomposites, photo-chemo therapy, targeted therapy

## Abstract

Treatment of malignancies on the chest wall, like chest wall recurrence of tumor, advanced cutaneous neoplasm and lymphoma, is still a challenge due to the involvement of the critical structures of heart and lung by the conventional strategy. The aim of the current study was to investigate targeted photo-chemo therapy mediated by Fe_3_O_4_@ZnO nanocomposites for malignancy on the chest wall while cardiopulmonary avoidance. Fe_3_O_4_@ZnO/Dox nanocomposites, the synthesis of the core-shell Fe_3_O_4_@ZnO nanocomposites followed by loading doxorubicin (Dox), were prepared to act as multifunctional drug delivery system (DDS). The synergistic anticancer effects on tumor on the chest wall and protection performance of heart and lung were evaluated in vitro and in vivo using cell viability assay, apoptosis detection, histopathologic examination, and serum biochemistry tests. Our observations demonstrated that Fe_3_O_4_@ZnO/Dox nanocomposites, could play the role of magnetic drug targeting to deliver Dox into tumor tissues and cells to enhance its chemotherapeutic efficiency. Besides, with ultraviolet (UV) illumination, Fe_3_O_4_@ZnO showed the excellent property of photosensitizer, further attacking the cancer cells by photodynamic therapy (PDT). Thus, apoptosis was synergistically induced by the photo-chemo therapy, resulting in a distinct improvement in anticancer activity. Since UV has a limited penetration distance in tissue, causing PDT to fail in the critical structures of heart and lung, cardiopulmonary hurt could be avoided during the treatment. Therefore, targeted photo-chemo therapy mediated by Fe_3_O_4_@ZnO nanocomposites may have promise as a potent treatment option for superficial malignancies on the chest wall while cardiopulmonary avoidance.

## INTRODUCTION

Due to the critical structures of heart and lung, treatment of malignancies on the chest wall, like chest wall recurrence of tumor, advanced cutaneous neoplasm and lymphoma, is still a challenge [1]. Conventional treatments consist of surgery, radiation, chemotherapy, or a combination of the above. Generally, wide local excision can be adequate for small superficial lesions. But, when the recurrence of tumor on the chest wall occurs, advanced cutaneous neoplasm and lymphoma, patients are not amenable to wide local excision with negative margins due to the bulk and extent of the tumor [2]. Meanwhile, radiotherapy to the chest wall could carry the risks of radiation-related toxicity such as pneumonitis, lung fibrosis, and coronary heart disease because considerable volumes of heart and lung are likely to receive high doses [2]. Although modern radiation therapy techniques have been introduced to avoid high dose exposure to surrounding and underlying healthy tissue, their clinical benefit is still a matter of debate [1]. Chemotherapy is usually obstructed mainly by low selectivity of the anticancer drugs towards the cancer cells, which causes severe side effects and systemic toxicity [3]. With that in mind, efforts should be made to develop new strategies of target coverage and cardiopulmonary sparing for malignancies on the chest wall.

Photodynamic therapy (PDT) is an approved new modality for the treatment of superficial solid tumor recurrent/refractory to conventional treatment based on photosensitizers exposed to light of specific wavelength [4, 5]. Among the photosensitizers, ZnO nanomaterials are regarded as the potential photosensitizing agents for PDT due to their unique phototoxic effect upon the irradiation [6–8]. With ultraviolet (UV) illumination, ZnO nanomaterials can generate cytotoxic reactive oxygen species (ROS) to kill tumor cells. Usually, the limitation of PDT to superficial malignant neoplasm is considered as its shortcoming since the UV light cannot penetrate deeply into human tissues [9–13]. However, just as fire is a good servant but a bad master, it is precisely the point of interest in the study. Thus, PDT mediated by ZnO nanomaterials exposed to UV illumination is especially desirable for superficial malignancy on the chest wall, since UV has a limited penetration distance in tissue, causing PDT to fail in the critical structures of heart and lung which are located far from the surface.

Multimodality treatment is a basic cancer treatment principle, and can enhance therapeutic outcomes [14–21]. Coincidentally, ZnO nanomaterials have emerged as ideal multimodal nanomedicine platforms, acting as not only drug carrier of Doxorubicin (Dox), a routine chemotherapeutic agent, to increase intracellular therapeutic agent, but also the photosensitizer in PDT of cancer, which were well illustrated in our previous studies [6, 7]. Thus, photo-chemo therapy mediated by ZnO nanomaterials, the combination of chemotherapy and PDT, has become an alternative to improve anticancer activity. However, the photo-chemo therapy is not specific for the target tissue. Thus, targeted drug delivery systems (DDS) are preferred, which have been used to increase the efficiency of drug delivery to specific tissues as well as to decrease its side effects [22–26]. One way to achieve it is by using magnetic carriers that allow the manipulation of pharmacodynamic properties using an external magnetic field [27, 28]. Magnetic drug targeting (MDT) is a novel solution for targeted delivery of drugs to tumors with minimal systemic toxicity, in which nanoparticles comprise of magnetic cores (such as Fe_3_O_4_) and shells loaded with drugs [29–31]. An external magnetic field applied at the tumor site localizes these core–shell particles at the tumor site enabling precise drug delivery.

To this end, Fe_3_O_4_@ZnO/Dox nanocomposites, the synthesis of the core-shell Fe_3_O_4_@ZnO followed by loading chemotherapeutic drug of Dox, were prepared in this study as multifunctional nanomedicine platforms for malignancy on the chest wall. In this case, the magnetic core of Fe_3_O_4_ upon an external magnetic field is applied for targeted delivery of chemotherapeutic drug of Dox to tumors. Moreover, the nature of PDT based on ZnO under UV illumination to superficial tumor further attacks the cancer cells, while sparing the critical structures of heart and lung. The in vitro and in vivo anticancer property and cardiopulmonary preservation of the targeted photo-chemo therapy were investigated in the xenograft model in which hepatocarcinoma SMMC-7721 cells were inoculated subcutaneously into the chest wall of the nude mice as the superficial malignancy.

## RESULTS AND DISCUSSION

### Structure and properties of the Fe_3_O_4_@ZnO nanocomposites

**Figure 1:**
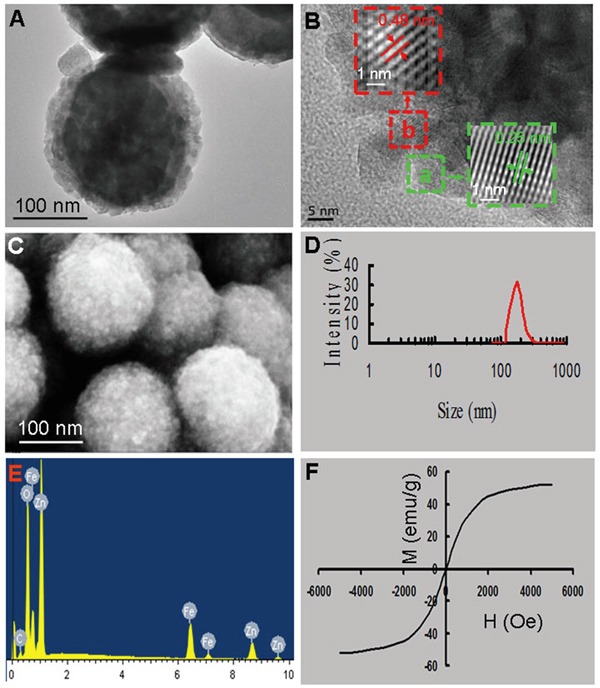
Characterizations of Fe_3_O_4_@ZnO nanocomposites **A.** TEM image. **B.** HRTEM and the corresponding reverse FFT of the HRTEM images taken from the areas. **C.** SEM image. **D.** Size distribution. **E.** EDS spectrum. **F.** Magnetization curves.

Biocompatible and multifunctional Fe_3_O_4_@ZnO nanocomposites could provide an innovative platform for cancer theranostics combined favorable magnetic targeting ability and photodynamic therapy [32–34]. In this study, the Fe_3_O_4_@ZnO nanocomposites were fabricated by the stepwise growth approach. Firstly, Fe_3_O_4_ nanoparticles were synthesized as nucleate seeds by coprecipitation of ferric and ferrous salt in alkaline medium. Secondly, the ZnO shell was growing on the surface of the cores by the addition of Zn(NO_3_)_2_, followed by a heating and aging process. From the transmission electron microscopy (TEM) image (Figure 1A), we can see that the synthesized Fe_3_O_4_@ZnO nanocomposites display the core/shell structure, consisting of the black Fe_3_O_4_ core and the light colored ZnO shell. The high resolution transmission electron microscopy (HRTEM) image, and the corresponding reverse fast Fourier transform (FFT) of the HRTEM images taken from the areas (marked with a green square for ZnO and a red square for Fe_3_O_4_) are shown in Figure 1B. It can be found that the lattice fringes and the corresponding lattice spacing are 0.25 nm and 0.48 nm, which are in good agreement with the (101) plane of ZnO and the (111) plane of Fe_3_O_4_, respectively. The scanning electron microscopy (SEM) image shows the homogeneous morphology (Figure 1C), and Figure 1D shows the size distribution of Fe_3_O_4_@ZnO determined by dynamic light scattering (Zetasizer, Malvern Instruments, UK). The mean size of the Fe_3_O_4_@ZnO is 183.98 ± 36.16 nm. The peaks corresponding to Fe, Zn and O are observed in the energy dispersive spectrometer (EDS) patterns (Figure 1E), further confirming the Fe_3_O_4_@ZnO nanocomposites were formed. The magnetization curve of Fe_3_O_4_@ZnO nanocomposites are drawn in Figure 1F, demonstrating that the samples were superparamagnetic because there was no hysteresis and both their remanence and coercivity were zero. Thus, Fe_3_O_4_@ZnO nanocomposites endow a promising application in biomedical fields including magnetic drug targeting and PDT.

### Fe_3_O_4_@ZnO nanocomposites as photosensitizers in PDT for cancer involved ROS upstream

**Figure 2:**
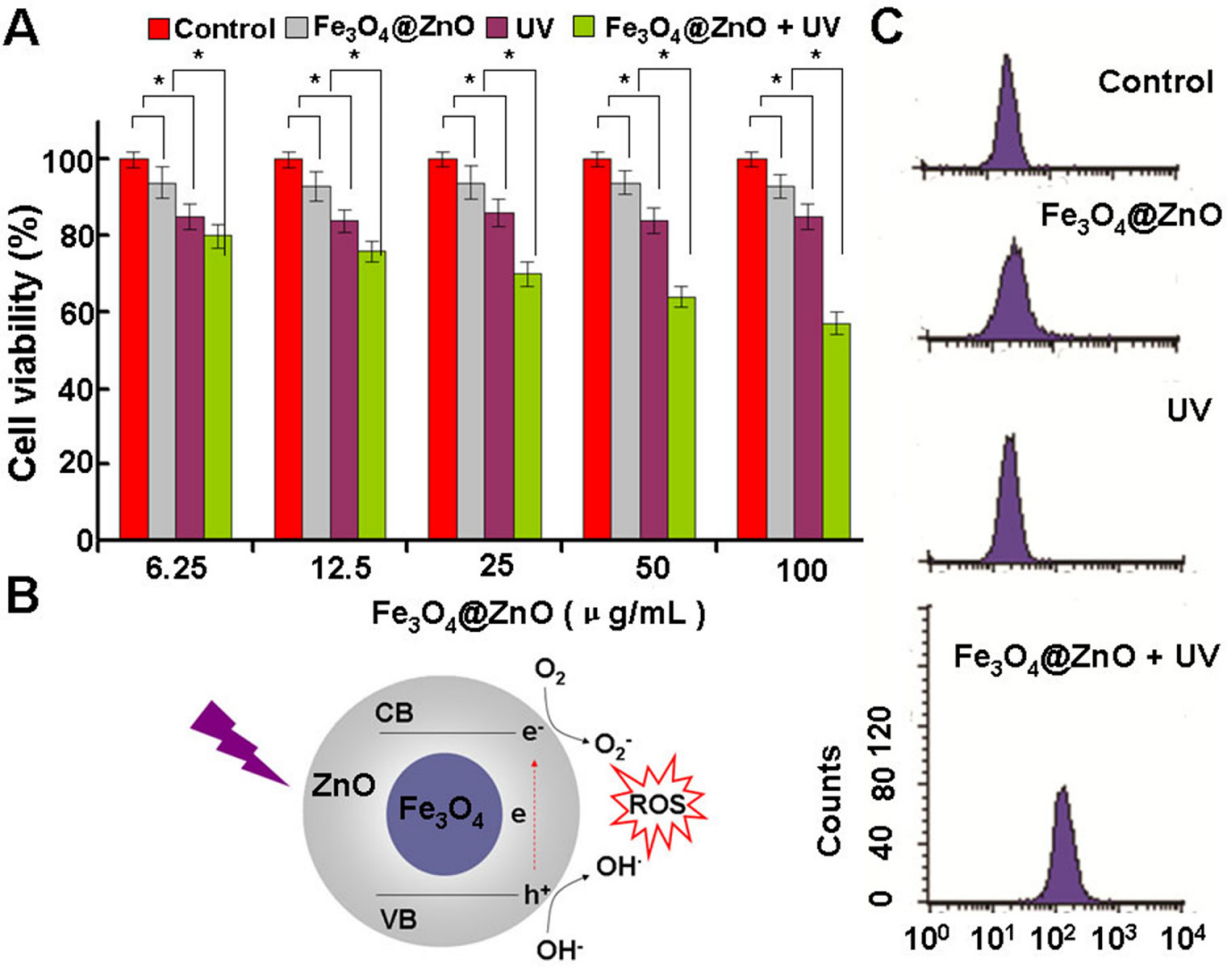
The photodynamic activity of the Fe_3_O_4_@ZnO nanocomposites **A.** SMMC-7721 cell viability treated with Fe_3_O_4_@ZnO nanocomposites in the absence and presence of UV irradiation. **B.** Scheme of mechanism of ROS production by Fe_3_O_4_@ZnO nanocomposites under UV irradiation. **C.** The intracellular production of ROS analyzed by the flow cytometer.

PDT is a promising new modality for the treatment of cancer because it promises a better selectivity for tumor that is accessible to light and low systemic toxicity with fewer side effects as compared to the radiation therapy and chemotherapy [4–8]. As evidently illustrated in Figure 2A, the lack of cytotoxicity of the Fe_3_O_4_@ZnO nanocomposites themselives against SMMC-7721 cells ensures a wide potential range of biomedical applications. The UV irradiation itself only showed a little cytotoxic effect on SMMC-7721 cells, whose surviving fraction was about 80%. However, when SMMC-7721 cells treated by Fe_3_O_4_@ZnO nanocomposites accompanied with UV irradiation, the cell viability decreased remarkably with a dose-dependent manner, which indicates that the photocatalytic activity of Fe_3_O_4_@ZnO nanocomposites under UV irradiation could promote mortality of cancer cells. ZnO, a semiconductor with band gap energy of 3.36 eV, will produce a hole (h^+^) in the valence band and an electron (e^−^) in the conduction band, namely the electron/hole pairs, under UV irradiation [6]. These electron/hole pairs will induce a series of photochemical reactions to generate ROS, due to the generation of hydroxyl radicals (OH^·^) by h^+^ abstracting electrons from water and/or hydroxyl ions and the production of the superoxide anion O_2_^−^ by e^−^ reducing O_2_, which is displayed in the Figure 2B. In this study, under UV irradiation, Fe_3_O_4_@ZnO nanocomposites could induced ROS within SMMC-7721 cells while there was no ROS production in the absence of UV irradiation and UV alone (Figure 2C). The formation of ROS could result in the cascade of cellular and molecular events relevant to tumor destruction, which is believed to be responsible for the promoting mortality of cancer cells [8].

### Magnet-guided targeted drug delivery and smart pH-responsive release of Fe_3_O_4_@ZnO/Dox nanocomposites

**Figure 3:**
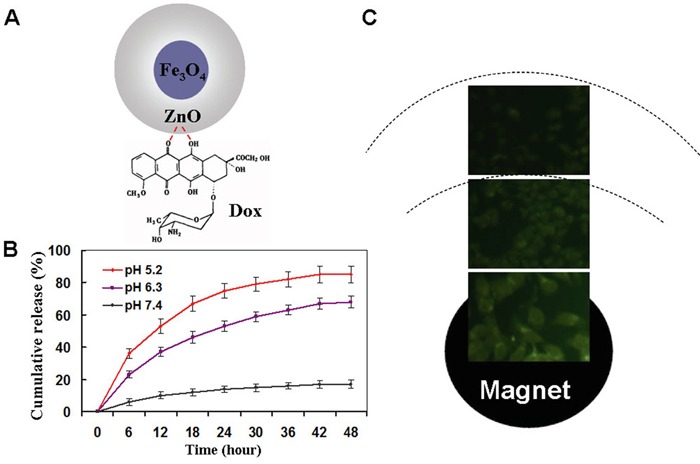
Magnetical targeted drug delivery and drug release property **A.** scheme for the Dox-loaded Fe3O4@ZnO nanocomposite. **B.** In vitro Dox release behavior at pH 7.4, 6.0, and 5.0. **C.** Fluorescence microscopy images of cells incubated with Fe_3_O_4_@ZnO/Dox nanocomposites right above the magnet and far from magnet.

Due to the metal binding sites of the quinone and the phenolic oxygen molecules on both sides of the Dox aromatic moiety, ZnO nanomaterials can act as a drug carrier to self-assemble Dox [6, 7, 35]. Figure 3A shows a proposed schematic representation of Dox loading onto Fe_3_O_4_@ZnO through the formation of Fe_3_O_4_@ZnO/Dox nanocomposites as a DDS. When Fe_3_O_4_@ZnO/Dox formed, the Zeta potentia decreased from – 18mV to −2 mV before and after the preparation, which strongly supported the fact that Dox was nicely combined with Fe_3_O_4_@ZnO nanocomposites. The encapsulation efficiency and loading efficiency were assessed and calculated to be 69.02± 4.83% and 19.71% ±2.36%, respectively. The release property of drug molecules was shown in Figure 3B, displaying pH and time depended manner. Dox release at lower pH of the tumor microenvironment, with approximately 65% (pH 6.3) and 83% (pH 5.2), was much faster than that about 16% at pH 7.4 of normal physiological conditions. The “smart” pH-triggered drug release behavior is favourite in practical cancer treatment, enabling more intelligent controlled release and enhancing chemotherapeutic efficiency [36]. We thus examined magnet-guided delivery of the Fe_3_O_4_@ZnO/Dox using a permanent magnet. The fluorescent Dox allowed an easy observation of cellular uptake of Fe_3_O_4_@ZnO/Dox by the SMMC-7721 cells through fluorescence microscopy without the need for additional markers. In our study, the optical microscopic observations of Dox fluorescence revealed the high uptake of Fe_3_O_4_@ZnO/Dox by the cancer cells above the magnet, with little Dox fluorescence seen in cells far from the magnet in the same culture dish after 6 h of incubation. These results clearly showed that Fe_3_O_4_@ZnO/Dox nanocomposites are better suited for magnetic induced targeted drug delivery in cancer therapy.

### In vitro photo-chemo therapy of Fe_3_O_4_@ZnO/Dox

**Figure 4:**
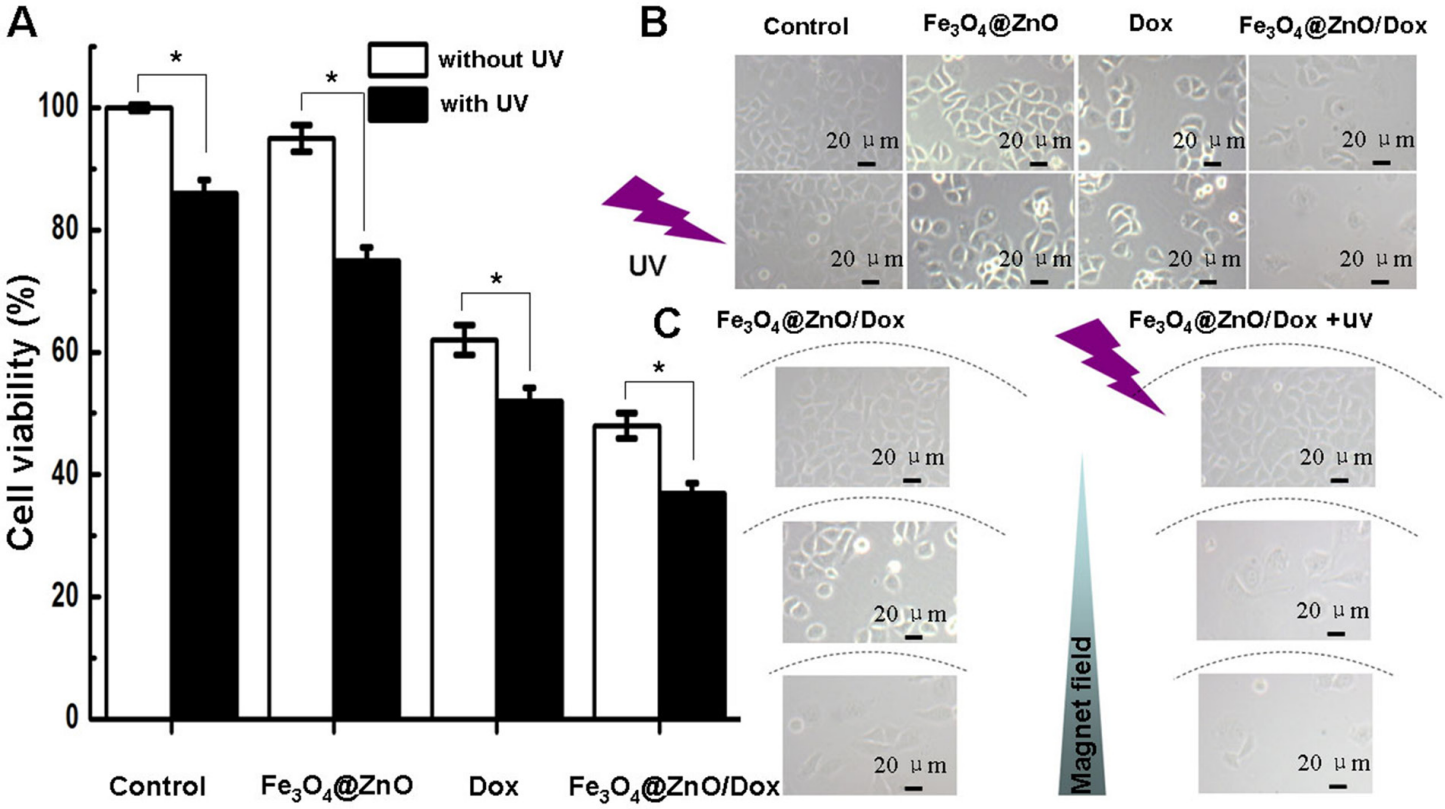
In vitro photo-chemo therapy of Fe_3_O_4_@ZnO/Dox **A.** Cytotoxic effect of Fe_3_O_4_@ZnO/Dox nanocomposites in the absence or presence of UV irradiation against SMCC-7721 cells. **B.** Microscopic images of SMCC-7721 cells after different treatments for 48 h. **C.** Microscopic images of the magnetically targeted photo-chemo therapy of Fe_3_O_4_@ZnO/Dox right above the magnet and far from magnet.

Since single modality has not always been sufficiently effective, combinations of cancer therapy modalities are attracting increasing attention to improve the outcome of treatment [6]. In the current study, as well illustrated above, Fe_3_O_4_@ZnO nanocomposites could not only be the photosensitizers for PDT but also play the role of the drug carrier for chemotherapy. To explore the possibility of the combination of PDT and chemotherapy, the anticancer effect of Fe_3_O_4_@ZnO/Dox nanocomposites were investigated as a strategy of photo-chemo therapy for human hepatocarcinoma SMMC-7721 cells. We cultured SMMC-7721 cells with Fe_3_O_4_@ZnO, Dox alone, or Fe_3_O_4_@ZnO/Dox (with equivalent Dox concentration of (0.5 μg/mL), with or without UV irradiation, for 48 h. Cells without any treatments were used as control. The cytotoxicity results were estimated by CCK-8 assay and shown in Figure 4A. Compared with those receiving Dox alone, the viability of the SMMC-7721 cells treated with Fe_3_O_4_@ZnO/Dox was decreased, especially Fe_3_O_4_@ZnO/Dox with UV irradiation. The obviously increased cytotoxicity attracted to the PDT activity of Fe_3_O_4_@ZnO promoting mortality of cells in addition to those mortality effects induced by Dox. The optical microscopic observations confirmed the results of CCK-8 assay (Figure 4B), which was obvious that Fe_3_O_4_@ZnO with UV irradiation could cause more significant morphological changes. The cancer cell morphology treated by the magnetically targeted Fe_3_O_4_@ZnO/Dox was shown in Figure 4C due to that CCK-8 assay could not be carried out in the same culture dish in vitro. The optical microscopic observations revealed that Fe_3_O_4_@ZnO/Dox under the magnetic field with UV irradiation could selectively kill cancer cells that were localized close to the magnet, without little change of cell morphology far from the magnetic field. Taken together, the synergistic effect of anticancer activity demonstrated that Fe_3_O_4_@ZnO/Dox could mediate targeted photo-chemo therapy for cancer.

### Photo-chemo therapy enhanced the growth suppression of malignancy on the chest wall in nude mice

**Figure 5:**
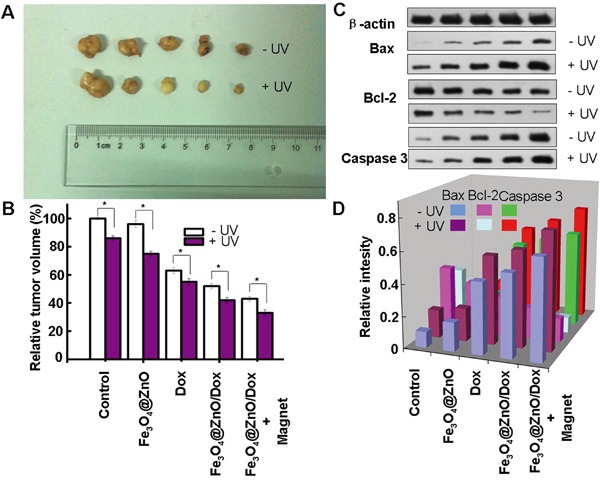
Targeted photo-chemo therapy mediated by Fe_3_O_4_@ZnO/Dox for metastatic tumor on the chest wall Appearance of tumor body at the end of the observation after 14 days treatment **A.** and relative tumor volume **B.** and the protein expression of Bax, Bcl-2 and Capase 3 **C.** and relative intensity of the protein expression after normalization by corresponding β-actin expression **D.**

Tumor recurrence is a major cause of morbidity and mortality in cancer [37], especially the recurrence of tumor on the chest wall due to the involvement of the critical structures of heart and lung [1]. Motivated by the above in vitro results, we then carried out in vivo investigation of targeted photo-chemo therapy of malignancy on the chest wall using the subcutaneous xenograft model while cardiopulmonary avoidance by Fe_3_O_4_@ZnO/Dox nanocomposites. After sacrificing the animals with different treatments at the end of the study for 14 days, the tumor appearance and the relative tumor volume were shown in Figure 5A and 5B. It was found that mice in the saline control group and treated by Fe_3_O_4_@ZnO showed severe tumour burden. Mice treated with Dox alone showed a brief reduction in tumor load. The Fe_3_O_4_@ZnO/Dox showed better inhibition effect than DOX alone at the equivalent dose. Compared with other groups, mice treated with Fe_3_O_4_@ZnO/Dox nanocomposites with the aid of magnet showed considerable slow down of tumor growth. This desired treatment effect might be attributed to magnetic drug targeting, allowing the DOX to accumulate selectively in the tumor tissues. In contrast, UV irradiation could clearly enhance suppression of tumor growth than that without UV irradiation, demonstrating that photo-chemo therapy mediated by Fe_3_O_4_@ZnO/Dox can promote tumor inhibition. Induced apoptosis is a popular anticancer strategy, which is the consequence of a series of precisely regulated events that are frequently altered in tumor cells, and resulted in the activation of caspases [7]. Caspase 3 is the most characterized effector caspase, and its activation leads to the final stages of cell death [6]. Therefore, we examined the changes in the protein expression levels of the apoptosis-regulating genes, including Bax, Bcl-2, and Caspase 3, to explore the possible signaling pathways through which Fe_3_O_4_@ZnO/Dox mediate photo-chemo therapy for metastatic tumor on the chest wall. As shown in Figure 5C and 5D, when mice were treated by Fe_3_O_4_@ZnO/Dox with the aid of magnet, the levels of Bax and Caspase 3 protein were significantly upregulated compared with other treatments. In contrast, UV irradiation could clearly enhance their expression than that without UV irradiation. Regarding to Bcl-2 protein, compared with other treatments, the levels in mice treated with Fe_3_O_4_@ZnO/Dox with the aid of magnet were significantly downregulated. What's more, UV irradiation could clearly decrease their expression than that without UV irradiation. Therefore, Fe_3_O_4_@ZnO nanocomposites could mediate targeted photo-chemo therapy of metastatic tumor on the chest wall.

### Cardiopulmonary avoidance

**Figure 6:**
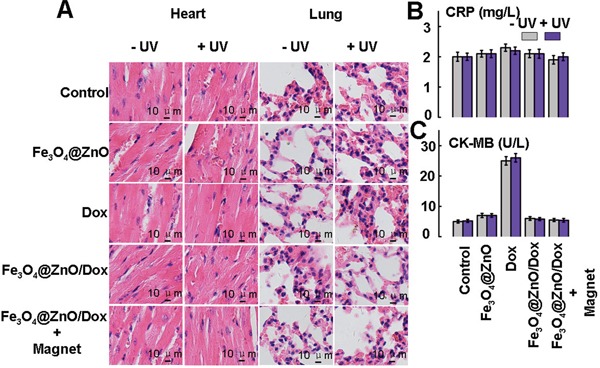
Histopathologic examination to determine the toxicity of heart and lung after different treatments at the end of the experiments **A.** Serum biochemistry data CRP for acute-phase response **B.** and CK-MB for cardiac function **C.**

The therapeutic management of malignancy on the chest wall often induces cardiopulmonary injury [38]. Surgery, even though conventionally considered minor, has been associated with the indirect induction of a pro-inflammatory profile [38]. Chemotherapy, such as Dox, the first line treatment for hematological malignancies and solid tumors due to broad spectrum of anticancer activity, is limited by its harmful side effects, especially its cardiotoxicity, which can lead to cardiomyopathy and congestive heart failure [39]. In addition, radiotherapy is associated with the development of pulmonary toxicity [1]. Therefore, effort should be made to improve anticancer efficiency while preserving normal tissue function. The aim of the present study was to investigate the photo-chemo property of Fe_3_O_4_@ZnO/Dox nanocomposites of the magnetic drug targeting and low penetration of UV photodynamic on superficial malignancy on the chest wall while sparing the critical structures of heart and lung. C-reactive protein (CRP) is a non-specific, acute-phase protein, primarily synthesized in the liver in response to cytokines, which be elevated in most forms of tissue damage, infection, inflammation [40]. CRP is a sensitive marker of the systemic inflammatory response [40]. Thus we quantitatively determined the levels of CRP to evaluate the cardiopulmonary injury. As shown in Figure 6A, there was no apparent histopathologic damage to heart and lung after different treatments. Meanwhile, the resulting serum biochemistry tests were shown in Figure 6B and 6C. There were also no apparent changes of CRP in all treatments. However, due to its cardiotoxicity, Dox alone may lead to rise of Creatine kinase MB isoenzyme (CK-MB), the indicators for cardiac function [41]. These indicate that the targeted photo-chemo therapy mediated by Fe_3_O_4_@ZnO/Dox nanocomposites could improve anticancer efficacy while avoiding its toxic side effects on normal tissues. The great performance makes it a promising candidate for superficial malignancy on the chest wall while cardiopulmonary avoidance.

**Figure 7:**
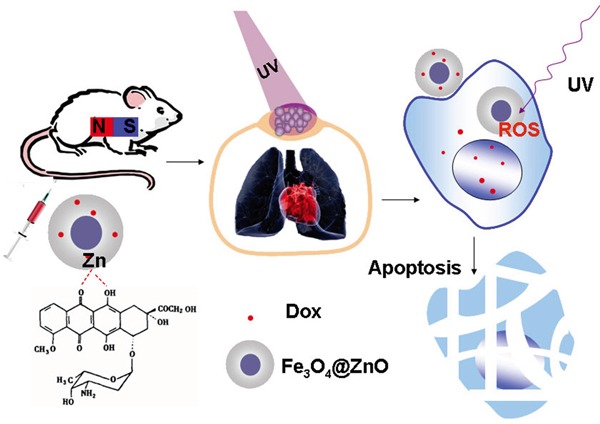
Schematic illustration of the process of Fe_3_O_4_@ZnO/Dox nanocomposites mediated targeted photo-chemo therapy of metastatic tumor on the chest wall while cardiopulmonary avoidance

Consequently, based on the above studies, Figure 7 schematically illustrates the possible processes of Fe_3_O_4_@ZnO/Dox mediated targeted photo-chemo therapy of malignancy on the chest wall while cardiopulmonary avoidance. Firstly, the synthesis of the core-shell Fe_3_O_4_@ZnO nanocomposites, followed by loading Dox, were prepared to act as multifunctional anticancer platform, in which Fe_3_O_4_@ZnO nanocomposites have the dual functions, i.e., not only the role of magnetic drug targeting carrier to deliver Dox into the targeted cancer cells and tissues, but also the photosensitizer under UV irradiation for the PDT of malignancy on the chest wall. Thereby, the increased intracellular drug enhanced the anticancer efficiency of chemotherapeutic agent. Moreover, the excellent photosensitizer activity of Fe_3_O_4_@ZnO nanocomposites further attack the cancer cells, while avoiding to hurt lung and heart due to low penetration of UV. Then the apoptosis, a preferred mode of killing the cancer cells, is induced synergistically by the photo-chemo therapy.

### Conclusions

In summary, in this study targeted photo-chemo therapy was designed for malignancy on the chest wall while cardiopulmonary avoidance based on Fe_3_O_4_@ZnO/Dox nanocomposites. The results demonstrated that Fe_3_O_4_@ZnO nanocomposites can delivery Dox into the targeted tumor cells and tissues with the aid of magnet to enhance its chemotherapeutic efficiency and reduce the side effect. In addition, Fe_3_O_4_@ZnO is as one of adjuncts to PDT under UV irradiation. Since UV has a limited penetration distance in tissue, causing PDT to fail in the critical structures of heart and lung, cardiopulmonary could be avoided to be hurt during the treatment. Therefore the Fe_3_O_4_@ZnO/Dox nanocomposites could present a promising strategy for malignancy on the chest wall, like chest wall recurrence of tumor, advanced cutaneous neoplasm and lymphoma, while cardiopulmonary avoidance.

## MATERIALS AND METHODS

### Chemicals

FeCl_2_·4H_2_O, FeCl_3_·6H_2_O, diethyleneglycol (DEG), Zn(NO_3_)_2_·6H_2_O, and NaOH were purchased from Shanghai Chemical Reagents Co (Shanghai, China). Dox was acquired from Hisun Phamaceuticals, Zhejiang, China. Cell Counting Kit-8 (CCK-8) and 2′,7′-dichlorofluorescin diacetate (DCFH-DA) were obtained from Sigma (St Louis, MO). RPMI-1640 culture medium, fetal bovine serum (FBS), and cultured plate were purchased from Gibco (Gibco/BRL, Carlsbad, CA). The antibodies of Bax, Bcl-2, Casepase 3, β-actin and horseradish peroxidase-conjugated IgG antibody were obtained from Nanjing KeyGen Biotech. Inc (Nanjing, China). All other reagents were of analytical grades.

### Self-assembled synthesis of the core-shell Fe_3_O_4_@ZnO nanoparticles

Initially, 2.0 mmol FeCl_2_·4H_2_O and 4.0 mmol of FeCl_3_·6H_2_O were dissolved in 50 mL DEG in a flask under protection with nitrogen gas, respectively. Meanwhile, 16 mmol of NaOH was dissolved in 25mL DEG. Then the solution of NaOH was added to a solution of metal chlorides with stirring at temperature 80°C, causing an immediate color change from bright yellow to deep green-brown. After reaction for 1 h, the temperature of the solution was raised to 200°C and then kept constant for 8 h in the temperature of 200°C in an autoclave. The solid product was isolated by cooling the reaction mixture to room temperature and centrifuging, and washed with ethanol five times and a mixture of ethanol and ethylacetate (1:1, v/v) five times to remove the excess of DEG. The above obtained Fe_3_O_4_ nanomaterial was dispersed in 50 mL of deionized water in a flask (250 mL). 2g Zn(NO_3_)_2_·6H_2_O was dissolved in 20 mL of deionized water and 1.48g tris was dissolved in 20 mL of deionized water. Then the solution of Zn(NO_3_)_2_ and tris were added to a solution of Fe_3_O_4_ with stirring at room temperature. At last, 0.4g PVP (Polyvinylpyrrolidone) was dissolved in 10 ml of deionized water and added to above solution, then the temperature slowly rose to 80°C and kept constant for 3 h in the temperature of 80°C. A precipitation was obtained and washed with ethanol five times and with deionized water five times.

### Characterization of Fe_3_O_4_@ZnO nanocomposites

The morphology of the as-synthesized Fe_3_O_4_@ZnO nanocomposites were observed by using transmission electron microscopy (TEM, Tokyo JEOL, Japan) and scanning electron microscopy (SEM, JEOL JSM-5900). The magnetization property at the room temperature of Fe_3_O_4_@ZnO nanocomposites in powder form was investigated by vibrating sample magnetometr (VSM, Model 7407, Lake Shore Cryotronics, Inc., OH, USA).

### In vitro the efficiency of Fe_3_O_4_@ZnO nanocomposites for cancer PDT

The human hepatocarcinoma SMMC-7721 cells, obtained from Shanghai Institute of cells, Chinese Academy of Sciences, were cultured in a RPMI 1640 medium supplemented with 10% FBS, 100 U/mL penicillin and 100 μg/mL streptomycin at 37°C in a humidified atmosphere of 5% CO_2_. The cells were treated with different concentrations of Fe_3_O_4_@ZnO nanocomposites (0, 6.25, 12.5, 25, 50, 100 μg/mL) for 48 h. UV irradiation (0.1 mW/cm^2^) was administrated by a UV lamp for 3 min at 6 h of cell culture. The relative cytotoxicity of Fe_3_O_4_@ZnO, UV irradiation, and Fe_3_O_4_@ZnO accompanied with UV irradiation were assessed with CCK-8 assays. Briefly, after cells with different treatments, CCK-8 (10 μL) were added to each well and incubated for additional 4 h. Optical density (OD) at 450 nm was recorded by the multi-well spectrophotometer reader (Thermo Labsystems, Vantta, Finland), then cell viability was calculated.

### ROS measurement

After a 6 h incubation of SMMC-7721 cells in the dark with Fe_3_O_4_@ZnO nanocomposites, the culture media was changed and photosensitized cells treated with or without UV irradiation, followed by addition of ROS probe of DCFH-DA into the cells for 20 minutes. Then, the cells were harvested by trypsinization and resuspended in PBS after washed for 3 times. The intracellular production of ROS was analyzed by the FACSCalibur flow cytometer, where the gate was arbitrarily set for the detection of green fluorescent DCF due to the oxidation of DCFH-DA by ROS.

### Preparation of Fe_3_O_4_@ZnO/Dox nanocomposites as drug delivery system

According to our previous study [6, 7], Fe_3_O_4_@ZnO/Dox nanocomposites were prepared as drug delivery system due to the metal binding sites of the quinone and the phenolic oxygen molecules on both sides of the Dox aromatic moiety. In the typical experiments, 2 mL aqueous solution of Dox (2 mg/mL) was added to a 1 mL aqueous suspension of the obtained Fe_3_O_4_@ZnO nanocomposites (10 mg/mL) in the dark overnight to construct Fe_3_O_4_@ZnO/Dox nanocomposites as drug delivery system. When the above DDS was collected by centrifugation, unbounded Dox in the supernatant were calculated by measuring the absorbance at 490 nm, allowing the estimation of the drug encapsulation and loading efficiency. In vitro magnetic targeting experiments were conducted by placing a round magnet under the center of the cell culture dish during incubation after Fe_3_O_4_@ZnO/Dox nanocomposites added into the cell culture. The cells were then observed by fluorescent microscopy (λex 488 nm, λem 515 nm) to visualize the cellular uptake of Dox. The release property of Dox was investigated at pH 7.4, 6.3 and 5.2 according to our previous study [6, 7]. Briefly, Fe_3_O_4_@ZnO/Dox nanocomposites were dispersed in PBS (pH 7.4, 5 mL) and transferred into a dialysis bag, which were then immersed in 95 mL PBS at pH 7.4, 6.3, or 5.2, and continuously agitated with a stirrer at 50 g and 37°C. At predetermined time intervals, 2 mL of the external medium was collected and replaced with the same fresh PBS. The amount of released Dox in the medium was then determined by high-performance liquid chromatography (LC-310; Skyray Instrument, Nanjing, China).

### Model of malignancy on the chest wall in nude mice

Four-week age BALB/c nude mice, weighing 16–22 g, from the Shanghai National Center for Laboratory Animals, were maintained in a specific pathogen-free facility. All the animal experiments were conducted under protocols approved by the animal ethics committee of the Medical School, Southeast University. 1×10^7^ human hepatocarcinoma SMMC-7721 cells suspended in 0.2 mL of RPMI-1640 medium were inoculated subcutaneously into the chest wall of the nude mice to establish the model of metastatic tumor on the chest wall. When the tumor size reached to ~50mm^3^, the tumor-bearing nude mice were assigned randomly and then intravenously administered via tail veins every three days with 200 mL of saline as control, Fe_3_O_4_@ZnO, Dox alone, Fe_3_O_4_@ZnO/Dox or Fe_3_O_4_@ZnO/Dox under the magnetic field, with or without UV irradiation. A magnet was gently placed in contact with the top of the tumor mass and held in the place for three hours to generate a magnetic field for drug delivery. This process was repeated every day for 14 days. When localized in the target tissue, Fe_3_O_4_@ZnO/Dox was activated with UV irradiation (300m J cm^2^) to produce ROS that destroy target tumor. On the 14^th^ day, the mice were killed, and the organs of heart and lung as well as the tumors were taken out, and the rate of tumor inhibition was calculated. To evaluate anticancer efficiency, two diameters of tumor were measured with digital calipers, and then tumor size was calculated using the following formulae: V = 1/2 × a × b^2^, where V is tumor volume, a is the longest diameter, and b is the shortest diameter. RTV (%) = V_test_/V_control_ × 100, where RTV is relative tumor volume at the end of experiment, V_control_ represents volume in control group, and V_test_ represents volume after different treatments.

### Histopathologic examination of tumor and organ tissue

After the mice were sacrificed, the organs of the heart and lung were quickly removed and fixed in 4% paraformaldehyde, dehydrated in a graded series of alcohol, and then embedded in paraffin. Tissue sections (4 μm thick) were prepared and stained with hematoxylin and eosin. Thereafter, the sections were examined by using Olympus IX51 microscope (400×; Olympus Corporation, Tokyo, Japan).

### Serum biochemistry tests

The blood was collected before the mice were sacrificed, and serum biochemistry tests of C-reactive protein (CRP) and Creatine kinase MB isoenzyme (CK-MB), were performed according to the manufacturer's instructions.

### Statistical analysis

All the data are presented as the mean ± standard deviation. The *F*-test was used for significance testing, and *P*<0.05 was considered to be statistically significant. All tests were performed using the Statistical Package for Social Science version 19.0 (SPSS Inc., Chicago, IL, USA).
